# Underweight, Stunting and Wasting among Children in Kilimanjaro Region, Tanzania; a Population-Based Cross-Sectional Study

**DOI:** 10.3390/ijerph14050509

**Published:** 2017-05-10

**Authors:** Melina Mgongo, Nikolas A. S. Chotta, Tamara H. Hashim, Jacqueline G. Uriyo, Damian J. Damian, Babill Stray-Pedersen, Sia E. Msuya, Margareta Wandel, Siri Vangen

**Affiliations:** 1 Institute of Clinical Medicine, University of Oslo, 0863 Oslo, Norway; sagumochotta@gmail.com (N.A.S.C.); jackieuriyo@yahoo.com (J.G.U.); babill.stray-pedersen@medisin.uio.no (B.S.-P.); sirvan@ous-hf.no (S.V.); 2 Better Health for African Mother and Child, P.O. Box 8418, Moshi, Tanzania; tammyhtz@yahoo.com (T.H.H.); siamsuya@hotmail.com (S.E.M.); 3 Department of Nutrition, Institute of Basic Medical Sciences, University of Oslo, P.O. Box 1046, 0317 Oslo, Norway; margareta.wandel@medisin.uio.no; 4 Institute of Public Health, Department of Community Health, Kilimanjaro Christian Medical University College, P.O. Box 2240, Moshi, Tanzania; d_jeremy5@yahoo.com; 5 Institute of Public Health, Department of Epidemiology and Biostatistics, Kilimanjaro Christian Medical University College, P.O. Box 2240, Moshi, Tanzania; 6 Division of Gynaecology and Obstetrics, Oslo University Hospital, Rikshospitalet, 0863 Oslo, Norway; 7 Norwegian National Advisory Unit for Women’s Health, 0863 Oslo, Norway

**Keywords:** underweight, stunting, wasting, breastfeeding, child illness, Kilimanjaro, Tanzania

## Abstract

This study assessed the prevalence and risk factors associated with underweight, stunting and wasting among children aged 0–24 months in six districts of Kilimanjaro region, northern Tanzania. A cross-sectional population-based study using a multistage, proportionate to size sampling was conducted from June 2010 to March 2011. A structured questionnaire was used to collect sociodemographic, economic, feeding and child information. Anthropometric data were collected by trained field workers, and the data were used to assess child nutritional status. A total of 1870 children were enrolled in this study. The prevalence of children classified as underweight was 46.0%, stunting was 41.9%, and wasting was 24.7%. About 33% were both underweight and stunted, and 12% had all three conditions. In a multivariate logistic regression, child age, child being ill and birth weight were associated with all anthropometric indices. Child being breastfed was associated with being underweight and wasting. Mother’s education was associated with being underweight and stunting. Fathers aged 35+ years, and living in the Hai district was associated with stunting, and being female was associated with wasting. The prevalence of child undernutrition is high in this region. Strategies that target each risk factor for child undernutrition may help to reduce the problem in the region.

## 1. Introduction

Child undernutrition in all its forms is a global health concern. Underweight, stunting and wasting are reported to be serious problems affecting developing countries [1]. Child undernutrition has short and long term effects. The short term effects include morbidity and mortality as it is reported to magnify the progression of disease and it contributes to 53% of deaths among children [2]. The long-term effects include preventing children from reaching their full developmental potential and poor cognitive performance, which in turn has consequences for the country’s productivity [1,3,4].

Global data shows that underweight caused 3.1 million deaths in 2011 [1]. In 2013, global data show that the prevalence of stunting, underweight, and wasting was 37%, 15% and 8%, respectively [5]. Undernutrition is reported to be higher in Asia and Africa than in Europe; in Africa the prevalence of stunting and underweight has increased for the past 23 years [5]. 

Tanzania has developed and implemented a number of programs in order to reduce child undernutrition such as infant and young child feeding, sanitation, deworming, vitamin A supplementation and health education [6]. Despite these programs, child undernutrition is still a challenge. The Tanzania Demographic and Health Survey (TDHS) has been following changes in nutritional status by using World Health Organization (WHO) Anthropometric indices to estimate the prevalence of undernutrition among children less than five years. These include weight for age (underweight), height for age (stunting) and weight for height (wasting) [7]. Underweight measures acute and chronic malnutrition, stunting indicates the status of chronic undernutrition in children, and wasting represents a failure to receive adequate nutrition in the period before the survey [5,7]. According to the TDHS, stunting increased from 38% in 2004/2005 to 42% in 2010, and then decreased in 2015 to 34% [6,8,9,10]. The TDHS provides national and regional data on child nutritional status, but data on specific districts are missing, and the TDHS does not assess the factors that affect child nutritional status at regional and district levels. This could affect the type of intervention introduced to overcome child undernutrition in specific districts. 

Factors that cause child undernutrition have been explained by the United Nations International Children’s Emergency Fund (UNICEF) conceptual framework of child malnutrition. Food intake or infection, or a combination of the two, are listed as major causes [7,11]. Other factors include poverty, low parental education, poor feeding practices, economic status, residence, family size, living in developing countries, number of under five children in one family, as well as urban and rural differences [9,12,13]. We have studies that have assessed child nutrition at the national level [8,14]. There is limited literature on nutritional status at district levels. This study was conducted to determine the developmental norms of children and the local risk factors for poor child development in the Kilimanjaro region [15]. Child nutrition was one of the risk factors that was assessed to determine child growth and development. This study was conducted in Kilimanjaro because we have missing information on child nutrition status at the district level. In the Kilimanjaro region, the districts are not homogenous in terms of health facilities and economic activities. This could have an impact on child nutritional status. Knowing the magnitude of the problem at the district level is an important aspect in coming up with strategies that can reduce the problem. This study therefore aimed to assess the prevalence and risk factors associated with underweight, stunting and wasting in the Kilimanjaro region of northern Tanzania.

## 2. Materials and Methods 

### 2.1. Study Design and Site

This population-based cross-sectional study was conducted from June 2010 to March 2011, involving mothers/caregivers with children aged 0–36 months in six districts of the Kilimanjaro region in Tanzania. This region has a population of 1,640,077, of whom 152,198 are children aged 0–36 months. The region has an annual population growth rate of 1.6% [16]. The largest part of this population lives in rural areas (75%) and depends on agriculture and livestock keeping. There is a wide coverage by health facilities that provide antenatal care, postnatal care and immunization.

### 2.2. Study Population

The study population consisted of families with children (age 0–36 months) in six districts of Kilimanjaro. The analysis for this paper was restricted to children aged 0–24 months.

### 2.3. Sampling Procedures

Multistage, proportionate to size sampling was used to select participants from six districts of the region. Village and street by single age population data from the 2002 census with 2009 projections were obtained from the Kilimanjaro regional bureau of statistics office in April 2009.

Initially, the populations of 0–36-month-old children for each village/street were listed with a column showing a cumulative population, which was used as a sampling frame. The sampling interval was then calculated: the total populations of 0–36-month-old children was divided by the required number of clusters, i.e., 50. A random number between 0 and 1 was generated from a computer and the starting point for selection of the first cluster was determined by multiplying the random number with the sampling interval. The subsequent cluster was located by adding the sample interval to the previous number until the 50th cluster was selected. Compact segment sampling was used to select households within the clusters. The selected clusters, i.e., enumeration areas, were mapped into segments with approximately equal populations. The number of segments was equal to the total population of 0–36-month-olds divided by the cluster size, i.e., 50. Each segment included 50 children. All segments were assigned a number on pieces of paper and one was randomly picked. Within the selected segment, the study team visited all the households until 50 children whose parents consented were examined. If all the households in a segment had been surveyed and less than 50 children were available, a second segment was randomly selected. The members of the households in the selected segments were requested to be available on the day of the survey.

### 2.4. Measures

#### 2.4.1. Sociodemographic and Economic Data

During interviews, a structured questionnaire was used to collect the mother’s sociodemographic information (age, marital status, years of education, occupation, number of live children), partner’s information (age, education, occupation) and socioeconomic status, and child information (age, sex, health condition, breastfeeding, growth and immunization). The study was approved by the Kilimanjaro Christian Medical Centre Ethical Committee (certificate number, 298), the National Institute for Medical Research (NIMR) Tanzania (Certificate number, 938), and the Norwegian Regional Ethical Committee (Certificate number, REK 2011/1068).

#### 2.4.2. Anthropometry

Anthropometric measurements (height and weight) were taken from the target child using recommended procedures [17]. The recumbent lengths for children less than 24 months of age were measured, and standing height was measured for older children. Weights of undressed children were taken using a SECA digital scale (SECA GmbH & Co. KG, Hamburg, Germany. The weight and height of children was taken to the nearest 0.1 kg and 0.1 cm respectively. Weight for age (WAZ), height for age (HAZ) and weight for height (WHZ) scores were generated using WHO standards [17,18]. All anthropometric measures were taken by two trained research assistants. Poor nutritional status was defined according to WHO set standards. Underweight, stunting and wasting were defined as having a Z score below −2SD [18].

#### 2.4.3. Haemoglobin Levels

A finger prick blood sample was collected from the target child and haemoglobin (Hb) levels were determined using the HaemoCue rapid testing method (HemoCue AB, Angelholm, Sweden). The hemoglobin analysis was carried out on site and the results were given to the child’s parent or guardian verbally. WHO cut off points to assess haemoglobin levels was used to assess anaemia. The cut off points for anaemia were: normal Hb levels ≥ 11.0 g/dL, mild anaemia Hb = 10.0–10.9 d/dL, moderate anaemia Hb = 7.0–9.9 g/dL and severe anaemia Hb < 7.0 g/dL [19].

#### 2.4.4. Data Analysis

**Figure 1:**
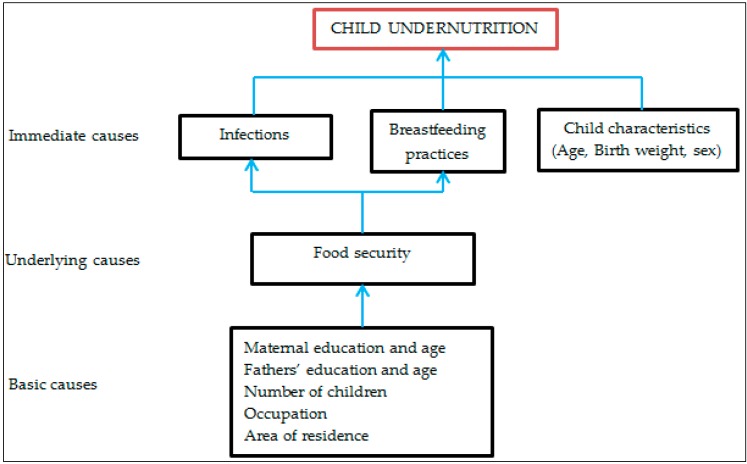
Modified conceptual framework for factors affecting child undernutrition.

Data were analyzed using Predictive Analytical Soft Ware (PASW) version 18. Descriptive statistics were used to summarize the data. The chi-squared test was used to test the significance of associations between dependent and independent variables. The dependent variables were underweight, stunting and wasting. The independent variables in this study were the mother’s age, area of residence, marital status, education level, number of children, employment, socioeconomic status, as well as sex of the child, birth weight, child health concern, born twins, breastfeeding and father’s education. Separate models were used to run each dependent variable and the independent variables. Logistic regression analysis was performed to control for confounders. Univariate logistic regression was performed, and all factors with a *p* value of <0.05 in the univariate analysis were included in the multivariate analysis model to obtain independent factors for underweight, stunting and wasting. Cases with missing values were excluded during the logistic regression. The clustering effect was considered during univariate and multivariate analyses. A multicollinearity test was performed before running the last model in the multivariate analysis and there was no evidence of correlation of independent variables. The conceptual framework (Figure 1) was used to determine the factors associated with child undernutrition. 

## 3. Results

In this study, there were 1870 observations and 47 clusters; the average cluster included 39.8 observations. The minimum number of observations in a cluster was two and maximum was 71. There was a significant difference between clusters with a *p* value of <0.009 and ρ of 1.8%.

### 3.1. Child Information

About 52% (*n* = 977) of the children in the study sample were males. The mean age of the children was 12.2 months (standard deviation, SD, 7.8), and 65.3% (*n* = 1213) of the children were anaemic (See Table 1).

### 3.2. Nutritional Status

Based on WHO definitions, 46.0% (*n* = 861) of the study sample were underweight, 41.9% (*n* = 784) stunted and 24.7% (*n* = 461) wasted. There was a significant difference of stunting among the six districts, *p* value < 0.001 (see Table 2).

In further analysis, 21.1% (*n* = 394) were both underweight and wasted, 12.1% (*n* = 226) were wasted and stunted and 32.5% (*n* = 607) were underweight and stunted. About 12% (*n* = 225) of children had all three nutritional status conditions.

### 3.3. Breastfeeding Practices

During exclusive breastfeeding (EBF) the child is given breastmilk alone for the first six months. EBF practice for infants aged 0–6 months was 22.7% (*n* = 413). EBF was not associated with underweight, stunting and wasting.

### 3.4. Factors Associated with Underweight, Stunting and Wasting

In a univariate logistic regression, the following factors were associated with underweight: mothers with primary education crude odds ratio (COR) (COR: 0.6, 95% confidence interval, CI: 0.5, 0.9) and secondary education (COR: 0.5, 95% CI: 0.3, 0.7), child aged 7–12 months (COR: 3.0, 95% CI: 2.4, 4.0), child aged 13–24 months (COR: 6.5, 95% CI: 5.0, 8.6), breastfeeding (COR: 0.4, 95% CI: 0.3, 0.5), child illness (COR: 2.6, 95% CI: 2.0, 3.7), high socioeconomic status (COR: 0.7, 95% CI: 0.5, 0.9). Other factors such as maternal age, number of live children, father’s education, child gender, number of children, mother’s occupation and residence were assessed, but were not associated with underweight. In a multivariate logistic regression, child aged 7–12 months adjusted odds ratio (AOR) (AOR: 3.1, 95% CI: 2.2, 4.3), child aged 13–24 months (AOR: 6.3, 95% CI: 4.5, 8.8), breastfeeding (AOR: 0.6, 95% CI: 0.4, 0.8), child illness (AOR: 2.0, 95% CI: 1.5, 2.7) and birth weight (AOR: 0.2, 95% CI: 0.1, 0.4) were the predictors of being underweight (Table 3). 

In a univariate logistic regression, the following factors were associated with stunting: mothers with primary and secondary education (COR: 0.6, 95% CI: 0.4, 0.9), father aged 35 years and above (COR: 0.6, 95% CI: 0.4, 0.9), child aged 7–12 months (COR: 1.2, 95% CI: 1.0, 1.6), child aged 13–24 months (COR: 3.1, 95% CI: 2.4, 4.0), breastfeeding (COR: 0.5, 95% CI: 0.4, 0.7), child illness (COR: 2.1, 95% CI: 1.7, 2.8), normal birth weight (COR: 0.2, 95% CI: 0.1, 0.3), living in Moshi District Council (COR: 0.6, 95% CI: 0.4, 0.9), living in Hai district (COR: 0.5, 95% CI: 0.3, 0.8). Other factors were assessed, but they were not associated with stunting. In a multivariate logistic regression, mothers with primary education (AOR: 0.6, 95% CI: 0.4, 0.9), mothers with secondary education and above (AOR: 0.6, 95% CI: 0.3, 0.9), father aged 35 years and above (AOR: 0.6, 95% CI: 0.4, 0.9), child aged 13–24 months (AOR: 2.9, 95% CI: 2.3, 3.9), child illness (AOR: 1.7, 95% CI: 1.3, 2.2), normal birth weight (AOR: 0.1, 95% CI: 0.1, 0.3), and living in Hai district (AOR: 0.5, 95% CI: 0.3, 0.8) were the independent predictors of stunting (Table 4).

In a univariate logistic regression, the following factors were associated with wasting: child aged 7–12 months (COR: 1.8, 95% CI: 1.4, 2.5), child aged 13–24 months (COR: 2.3, 95% CI: 1.7, 3.0), breastfeeding (COR: 0.5, 95% CI: 0.4, 0.7) and child illness (COR: 2.4, 95% CI: 1.9, 3.1).

In a multivariate logistic regression, child aged 7–12 months (AOR: 1.9, 95% CI: 1.3, 2.6), child aged 13–24 (AOR: 1.9, 95% CI: 1.4, 2.7), child being female (AOR: 0.8, 95% CI: 0.6, 0.9) and child illness (AOR: 2.3, 95% CI: 1.7, 3.0) remained associated with wasting, as outlined in Table 5.

## 4. Discussion

This paper describes the child nutrition situation in six districts of Kilimanjaro and highlights the burden of more than one coexisting undernutrition conditions among children. This study showed a high prevalence of underweight (46.0%), stunting (41.9%) and wasting (24.7%). In further analysis, 33% of children were both stunted and underweight, 21% were underweight and wasted, and 12% were stunted and wasted. Factors that were associated with child undernutrition included the child being sick, child’s age, birth weight, maternal education, being female, breastfeeding, living in the Hai district and the father’s age.

The prevalence of child undernutrition is high in this setting. Other researchers in East Africa have reported an even higher prevalence of child undernutrition [14,20,21]. The prevalence reported in this study is higher compared to the one reported by the Tanzania Demographic and Health Survey 2010/2015. We observed a high prevalence of underweight. Underweight is reported to increase the risk of under-five death, as well as result in a greater risk of infection and a slow recovery from illness [22]. Our results also show a high prevalence of stunting. Stunting is reported by other researchers to be a common health problem in Tanzania [23,24]. The TDHS shows that, for a period of 10 years, stunting was reduced by 8%. This is slow progress in reducing the stunting rate among children. According to Sustainable Development Goal (SDG) number 3, the global stunting rate should be reduced by 40% by the year 2025. If we fail to reduce the stunting condition, children will be exposed to the long term effects of stunting and may not reach their full growth potential [25,26]. Stunting is reversible during the first 1000 days of an infant’s life. Beyond that, it is irreversible [1]. Interventions that aim to promote maternal and child health at this period may reduce the problem of undernutrition and poor growth outcome [25]. Specific interventions to target local factors influencing stunting in this region may help to reach the SDG goal by 2025. 

In this study, we observed that 33% of children both stunted and underweight and 12% are living with all three conditions. These children are at a higher risk of developmental delays and poor cognitive performance, leading to poor performance at school and, later in life, poor socioeconomic status and hence a continued vicious circle of poor nutrition. SDG 3 encourages the country to promote the well-being of their citizens with a special focus on early childhood [27]. This may help to break the vicious circle of stunting in our communities. There is a need for more efforts to reduce child undernutrition. 

Maternal education reduced the odds for a child to be underweight and stunted. Maternal education is reported by other researchers to have a protective effect against child undernutrition [14,19]. Maternal education might be an essential factor in proper infant feeding practices. Educated mothers might also have a better income.

Child illness increased the odds ratio of the child being underweight, wasted and/or stunted. Several studies have shown that child illness has a negative effect on child growth [28,29]. Child illness affects dietary intake, absorption and utilization of nutrients, and hence affects child nutritional status [11,30]. It is also important to treat infections early to avoid the risks of child undernutrition. As our study was cross-sectional (data were collected at one point), we cannot say which was the initiating factor, infections or undernutrition. 

Child age was associated with underweight, stunting and wasting. In this study results showed as the child’s age increased, the odds ratio of being underweight increased. Similar findings were reported by the TDHS [8,9]. Several studies show that an increase in child age is a risk for undernutrition [16,21,31]. A study by Marriot also reported a higher prevalence of undernutrition among children aged 12–23 months compared to the younger age group [31].

In this study, about 23% of children were exclusively breastfed and 84% of children were still breastfed. EBF had no effect on child undernutrition, but children who were breastfed had decreased odds of being underweight. Studies in Ethiopia and Botswana showed that breastfeeding reduced the odds of a child being underweight and wasted [29,32]. The results of this study conflict with the results of the longitudinal study conducted by Fawzi and colleagues, which showed that prolonged breastfeeding was not a protective factor against underweight. In fact, it showed that prolonged breastfed children from poorer and illiterate mothers had a higher risk of being undernourished [33]. Despite the conflicting results, the results of our study support the WHO recommendation that children should be breastfed up to two years or beyond, because breastfeeding has been shown to have other protective effects on child health. 

In this study, socioeconomic status was not associated with child undernutrition. Other studies showed that the economic status of a family plays a big role in child nutrition. Poor economic status may limit the family’s to access food and health services. The TDHS report showed that children from a lower economic status had a greater risk of being underweight than those of a higher status [8]. A study in Tamil Nadu showed that children from families with a higher economic status had decreased prevalence of undernutrition [34]. Moreover, it is well documented that the burden of undernutrition affects the poor [13,35,36].

In this study, female children had lower odds for being wasted. Several studies have shown that males were more at risk of being undernourished than females [8,32,33,34,37]. 

Children with normal birth weight had reduced odds of being stunted, wasted or underweight. Other studies have reported the protective effect of normal birth weight on child nutritional status [32,38]. Low birth weight is caused by poor maternal nutrition during conception or pregnancy [39]. Studies show that interventions such as micronutrient supplementation among pregnant women helps in reducing the risk of low birth weight [40].

The strength of this study lies in the fact that we collected data from six districts of Kilimanjaro, so the results of this study can be generalized in the six districts. We also used a large sample size to estimate the prevalence of underweight, stunting and wasting. This study is limited in its design. We used a cross-sectional design, so our study cannot indicate a causal relationship of the factors associated with underweight, wasting and stunting.

## 5. Conclusions

The results of our study point to the need of a multi-sectoral approach to target child undernutrition among children less than two years of age. Broad approaches targeting mother’s education and women’s socioeconomic status are needed. This may be helpful to reduce the problem of child undernutrition. There is also a need for interventions to promote health care seeking and the treatment of childhood infections, as well as maternal health and nutrition during pregnancy to reduce low birth weight in children.

## Figures and Tables

**Table 1:** Background characteristics of children, N = 1870.

Variable Name	N	%
**Child age (months)**		
0–6	482	23.9
7–12	649	32.2
13–24	739	36.7
**Sex**		
Male	977	52.2
Female	893	47.8
Anaemia status *		
Normal	646	34.8
Anaemic	1213	65.3
**Residence**		
Urban	623	33.3
Rural	1247	66.7

* variable with missing information.

**Table 2:** Description of nutritional status in relation to child age, sex and districts, N = 1870.

Variable Name	N	Stunted *n* (%)	*p* Value	Underweight *n* (%)	*p* Value	Wasted *n* (%)	*p* Value
**Age (months)**							
0–6	482	145 (30.1)	<0.001	104 (21.6)	<0.001	77 (16.0)	<0.0001
7–12	649	255 (34.7)		291 (44.8)		166 (25.6)	
13–24	739	414 (56.0)		466 (63.1)		218 (29.5)	
**Sex**							
Male	977	416 (42.6)	0.549	466 (47.7)	0.133	260 (26.6)	0.040
Female	893	368 (41.2)		395 (44.2)		201 (22.5)	
**Districts**							
Mwanga	168	82 (48.8)	<0.001	79 (47.2)	0.116	34 (20.2)	0.144
Rombo	470	231 (49.2)		234 (49.8)		106 (22.55)	
Moshi District Council	582	220 (37.8)		252 (43.3)		143 (24.6)	
Moshi Municipal Council	230	97 (42.2)		93 (40.3)		55 (23.9)	
Hai	225	74 (32.9)		105 (46.7)		63 (28.0)	
Siha	195	80 (41.0)		98 (50.3)		60 (30.8)	

**Table 3:** Factors associated with underweight, N = 1870.

Variable Name	N	*n* (%) Underweight	COR (95% CI)	*p* Value	AOR (95% CI)	*p* Value
**Mother’s education**						
None/primary incomplete	138	80 (58.0)	1		1	
Primary complete	1542	709 (46)	0.6 (0.4, 0.9)	0.012	0.7 (0.4, 1.1)	0.136
Secondary and above	190	72 (37.9)	0.5 (0.3, 0.7)	0.001	0.5 (0.3, 0.9)	0.039
**Child information**						
Child’s age 0–6	482	104 (21.6)	1		1	
7–12	649	291 (44.8)	3.0 (2.3, 4.0)	<0.001	3.1 (2.2, 4.3)	<0.001
13–24	739	466 (63.1)	6.5 (5.0, 8.6)		6.3 (4.5, 8.8)	
**Sex**						
Male	977	466 (47.8)	1		1	
Female	893	395 (44.2)	0.9 (0.7, 1.0)	0.128	0.9 (0.7, 1.1)	0.190
Birth weight *						
Low birth weight	98	75 (76.5)	1		1	
Normal	1705	749 (43.9)	0.2 (0.1, 0.4)	<0.001	0.2 (0.1, 0.4)	<0.001
Breastfeeding *						
No	287	180 (62.7)	1			
Yes	1540	656 (42.6)	0.4 (0.3, 0.5)	<0.001	0.6 (0.4, 0.8)	0.001
Child illness *						
No	1500	623 (41.5)	1		1	
Yes	360	233 (64.7)	2.6 (2.0, 3.3)	<0.001	2.0 (1.5, 2.7)	<0.001
**SES**						
Low	500	251 (50.2)				
Medium	505	238 (47.1)	0.9 (0.7, 1.1)	0.248	0.9 (0.6, 1.2)	0.333
High	487	208 (42.7)	0.7 (0.5, 0.9)	0.016	0.8 (0.6, 1.0)	0.073

Mother’s education, child’s age in months, sex, birth weight, breastfeeding, child illness and socioeconomic status were variables adjusted in the model; low birth weight: child born weighing less than 2500 g; * variables with missing information; AOR: adjusted odds ratio; CI: confidence interval; COR: crude odds ratio; SES: socioeconomic status.

**Table 4:** Factors associated with stunting, N = 1870.

Variable Name	N	*n* (%) Stunted	COR	*p* Value	AOR	*p* Value
**Mother’s education**						
None/primary incomplete	138	73 (52.9)	1		1	
Primary complete	1542	636 (41.3)	0.6 (0.4, 0.9)	0.008	0.6 (0.4, 0.9)	0.025
Secondary and above	190	75 (39.5)	0.6 (0.4, 0.9)	0.020	0.6 (0.3, 0.9)	0.043
Father’s age *						
15–24	167	78 (46.7)	1		1	
25–34	759	325 (42.8)	0.8 (0.6, 1.2)		0.8 (0.5, 1.2)	
35+	755	282 (37.5)	0.7 (0.5, 0.9)	0.029	0.6 (0.4, 0.8)	0.008
**Child information**						
Child’s age 0–6	482	145 (30.1)	1		1	
7–12	649	255 (34.7)	1.2 (1.0, 1.6)	0.090	1.2 (0.9, 1.6)	0.233
13–24	739	414 (56.0)	3.1 (2.4, 4.0)	<0.001	2.9 (2.3, 3.9)	<0.001
**Sex**						
Male	977	416 (42.6)	1		1	
Female	893	368 (41.2)	0.9 (0.8, 1.1)	0.498	0.8 (0.6, 1.0)	0.047
Birth weight *						
Low birth weight	98	77 (78.6)	1		1	
Normal	1705	676 (39.7)	0.2 (0.1, 0.3)	<0.001	0.2 (0.1, 0.3)	<0.001
**Breastfeeding**						
No	287	160 (55.8)	1			
Yes	1540	601 (39.0)	0.5 (0.4, 0.7)	<0.001	0.7 (0.5, 1.0)	0.047
Child illness *						
No	1500	573 (38.2)				
Yes	360	205 (56.9)	2.1 (1.7, 2.8)	<0.001	1.7 (1.3, 2.2)	<0.001
**Districts**						
Mwanga	168	82 (44.8)	1		1	1
Rombo	470	231 (49.2)	1.0 (0.8, 1.6)	0.939	1.2 (0.7, 1.8)	0.595
Moshi District council	582	220 (37.8)	0.6 (0.4, 0.9)	0.012	0.7 (0.4, 1.1)	0.059
Moshi Municipal council	230	97 (42.2)	0.7 (0.5, 1.2)	0.201	0.7 (0.4, 1.1)	0.113
Hai	225	74 (32.9)	0.5 (0.3, 0.8)	0.002	0.5 (0.3, 0.8)	0.006
Siha	195	80 (41.0)	0.7 (0.5, 1.1)	0.148	0.7 (0.4, 1.2)	0.231
**SES**						
Low	500	226 (45.2)	1			
Medium	505	211 (41.8)	0.9 (0.7, 1.1)	0.243		
High	487	191 (39.2)	0.8 (0.6, 1.1)	0.060		

Mother’s education, father’s age, child’s age, sex, birth weight, breastfeeding, child illness, and districts were variables adjusted in the model; low birth weight: child born weighing less than 2500 g; * variables with missing information.

**Table 5:** Factors associated with wasting, N = 1870.

Variable Name	N	*n* (%) Wasted	COR	*p* Value	AOR	*p* Value
**Child information**						
Child’s age 0–6	482	77 (16.0)	1		1	
7–12	649	166 (25.6)	1.8 (1.4, 2.5)	<0.01	1.9 (1.3, 2.6)	<0.001
13–24	739	218 (29.5)	2.3 (1.7, 3.0)	<0.01	1.9 (1.4, 2.7)	<0.001
**Sex**						
Male	977	260 (26.6)	1		1	
Female	893	201 (22.5)	0.8 (0.6, 1.0)	0.038	0.8 (0.6, 0.9)	0.015
Birth weight *						
Low weight	98	36 (36.7)	1		1	
Normal	1705	400 (23.5)	0.5 (0.3, 0.8)	0.004	0.5 (0.3, 0.8)	0.005
Breastfeeding *						
No	287	99 (34.5)	1		1	
Yes	1540	351 (22.8)	0.5 (0.4, 0.7)	<0.001	0.6 (0.5, 0.9)	0.006
Child illness *						
No	1500	316 (21.1)	1		1	
Yes	360	142 (39.4)	2.4 (1.9, 3.1)	<0.01	2.3 (1.7, 3.0)	<0.001
**SES**						
Low	500	126 (25.2)	1			
Medium	505	121 (24.0)	0.9 (0.7, 1.2)	0.598		
High	487	120 (24.6)	1.0 (0.7, 1.3)	0.773		

Child’s age, sex, birth weight, current breastfeeding, and child illness were variables adjusted in the model; * variables with missing information.
